# TAp63γ Demethylation Regulates Protein Stability and Cellular Distribution during Neural Stem Cell Differentiation

**DOI:** 10.1371/journal.pone.0052417

**Published:** 2012-12-14

**Authors:** Maria B. Fonseca, Ana F. Nunes, Ana L. Morgado, Susana Solá, Cecília M. P. Rodrigues

**Affiliations:** 1 Research Institute for Medicines and Pharmaceutical Sciences (iMed.UL), Faculty of Pharmacy, University of Lisbon, Lisbon, Portugal; 2 Department of Biochemistry and Human Biology, Faculty of Pharmacy, University of Lisbon, Lisbon, Portugal; Università degli Studi di Milano, Italy

## Abstract

p63 is a close relative of the p53 tumor suppressor and transcription factor that modulates cell fate. The full-length isoform of p63, containing a transactivation (TA) domain (TAp63) is an essential proapoptotic protein in neural development. The role of p63 in epithelial development is also well established; however, its precise function during neural differentiation remains largely controversial. Recently, it has been demonstrated that several conserved elements of apoptosis are also integral components of cellular differentiation; p53 directly interacts with key regulators of neurogenesis. The aim of this study was to evaluate the role of p63 during mouse neural stem cell (NSC) differentiation and test whether the histone H3 lysine 27-specific demethylase JMJD3 interacts with p63 to redirect NSCs to neurogenesis. Our results showed that JMJD3 and TAp63γ are coordinately regulated to establish neural-specific gene expression programs in NSCs undergoing differentiation. JMJD3 overexpression increased TAp63γ levels in a demethylase activity-dependent manner. Importantly, overexpression of TAp63γ increased β-III tubulin whereas downregulation of TAp63γ by specific p63 siRNA decreased β-III tubulin. Immunoprecipitation assays demonstrated direct interaction between TAp63γ and JMJD3, and modulation of TAp63γ methylation status by JMJD3-demethylase activity. Importantly, the demethylase activity of JMJD3 influenced TAp63γ protein stabilization and cellular distribution, as well as TAp63γ-regulated neurogenesis. These findings clarify the role of p63 in adult neural progenitor cells and reveal TAp63γ as a direct target for JMJD3-mediated neuronal commitment.

## Introduction

The transcription factor p63, member of the p53 family, can be expressed as a full-length isoform containing a transactivation (TA) domain, termed TAp63, or as a truncated isoform that lacks the TA domain, termed ΔNp63. This isoform functions, at least in part, as naturally occurring dominant-inhibitor of full-length p53 family members. Additionally, both TAp63 and ΔNp63 undergo C-terminal alternative splicing giving rise to at least 6 different isoforms, which along with post-translational modifications (PTM), accounts for molecular and regulatory complexity [1]–[3]. Full-length p53 family members show structural and functional similarities, including a comparable domain structure of an N-terminal TA domain, an invariant DNA-binding domain, and an oligomerization domain [4]. p63 has been well-established as an important regulator of cell survival and cell death in the nervous system, primarily by acting through the apoptosis machinery [2], [3], [5]. In this respect, we have shown that both TA and ΔNp63 isoforms are involved in molecular mechanisms associated with Alzheimer's disease [3].

Recently, it has been suggested that cell death-relevant proteins, especially those involved in the core of the executing apoptosis machinery, may play a dual function in differentiation and cell death [6]. Restrictive activation and careful regulation will assure differentiation efficiency and thus avoid cell loss. In this regard, we have previously demonstrated the involvement of specific apoptosis-related microRNAs and proteins, including p53, caspases and calpains, in mouse neural stem cell (NSC) differentiation, by mechanisms that do not result in cell death [7]–[11]. Several studies have shown that inactivation of p53 sustains the undifferentiated state of neural precursor cells [12]–[14], and that p53 is required for both neurite outgrowth and axonal regeneration in mice primary neurons [15]. We have further dissected the role of p53 during NSC differentiation by showing that this proapoptotic protein cross-talks with key regulators of neurogenesis, such as the histone H3 lysine 27-specific demethylse JMJD3 [9]. In fact, PTM of apoptosis-related proteins may redirect stem cells to differentiation, as an alternative to cell death, and establish tissue-specific programs of gene expression throughout differentiation. A strong interplay between p53, p63 and p73 isoforms has been recently demonstrated, which orchestrates cell fate decisions [16], [17]. In addition, similar to p53, TAp63 can oligomerize, bind to DNA, transactivate p53 target genes, and induce cell cycle arrest and apoptosis [1], [3], [17].

The pivotal role of p63 during epithelial development has been well recognized. Mice lacking all p63 isoforms die at birth and show severe developmental abnormalities, including limb truncation, and defects in the epidermis and its appendages [18]. However, although TAp63 critical function in sympathetic neuronal development is firmly established [5], two independent studies have recently reported that p63 is not essential for central nervous system development [19], [20]. Nevertheless, in contrast to p63-restricted expression observed during mice and human brain development, high levels of p63 protein and mRNA were observed in adult human cerebral cortex and hippocampus [19], suggesting a role for p63 in the adult brain. Furthermore, in postnatal mice, p63 was shown to be expressed in neurogenic niches, such as the subventricular zone of the lateral ventricle [19]. Together, this evidence supports the idea that p63 may have a key role during adult neurogenesis.

Here, we investigated p63 involvement during adult mouse NSC differentiation and explored the potential cross-talk between p63 and JMJD3 in this cellular context. Our results demonstrated that TAp63γ and JMJD3 are coordinately regulated during NSC differentiation to establish a neuronal-specific gene expression program. In addition, TAp63γ subcellular distribution and neurogenic functions may be regulated by direct JMJD3-dependent demethylation of TAp63γ.

## Materials and Methods

### Ethics Statement

The mouse NSC line used in this study was obtained from Dr. Smith's Laboratory, University of Cambridge, Cambridge, UK [9], [21], [22], and provided by Dr. Henrique, University of Lisbon, Lisbon, Portugal. The Animal Ethical Committee at the Faculty of Pharmacy, University of Lisbon, Portugal waived the need for approval.

### Cell Culture and Differentiation

A mouse NSC line was derived from day 14.5 post-coitum mouse fetal forebrain [9], [21], [22]. This cell line was established using a method that produces pure cultures of adherent NSCs, which continuously expand by symmetrical division, and are capable of tripotential differentiation [23]–[25]. Cells were grown in monolayer as previously described [11], [26] and routinely maintained in undifferentiation medium, Euromed-N medium (EuroClone S.p.A., Pavia, Italy), supplemented with 1% N-2 supplement (Invitrogen Corp., Grand Island, NY), 20 ng/mL epidermal growth factor (EGF; PeproTech EC, London, UK), 20 ng/mL basic fibroblast growth factor (bFGF; PeproTech EC) and 1% penicillin-streptomycin (Invitrogen Corp.), in uncoated tissue culture plastic flasks at 37°C in a humidified atmosphere of 5% CO_2_. Medium was changed every 3 days and cells collected with accutase (Sigma-Aldrich Co., St. Louis, MO) when confluent. Differentiation of NSCs was performed by first platting cells in undifferentiation medium onto uncoated tissue culture plastic dishes at 1×10^6^ cells/cm^2^ for 24 h, and changing the culture medium to differentiation medium, Euromed-N medium supplemented with 10 ng/mL bFGF, 0.5% N-2 supplement, 1% B27 supplement and 1% penicillin-streptomycin. Cells were collected before medium change (time 0), or cultured for additional 6, 12, 24 or 48 h, and then fixed for immunocytochemistry analysis or collected for protein extraction.

### siRNAs and Plasmid Transfections

The NSC cell line was transfected with Flag-JMJD3 or Flag-JMJD3 mutant overexpression plasmids to amplify JMJD3 expression. The expression plasmids were kindly provided by Dr. Kristen Jepsen (University of California, San Diego, USA). The Flag-JMJD3 construct was cloned by inserting full-length mouse JMJD3 cDNA in frame into p3xFLAG CMV-10 (Sigma-Aldrich Co.) vector within HindIII and BamHI sites. The Flag-JMJD3 mutant was generated by removing the carboxy-terminus 410 amino acids, which include the jumonji C domain. Briefly, NSCs were first cultured in uncoated dishes in undifferentiation medium without penicillin-streptomycin. Twenty-four hours after plating, cells were transfected using Lipofectamine 2000 (Invitrogen Corp.), according to the manufacturer's instructions, the medium was changed to differentiation medium, and cells were cultured for additional 24 h. The cDNAs for TAp63γ were ligated as BamHI/XhoI fragments into the pcDNA3.1/His C vector (Invitrogen Corp.). Mutant TAp63γ carries a R304H exchange to yield a mutation in the DNA binding domain, as previously described [27]. Briefly, the mutant expression vector includes a His at the equivalent Arg position, which is transcriptionally impaired. As a control (mock), cells were incubated with the transfection agent at the same concentration and time, in the absence of any plasmid. Attached cells were either harvested for immunobloting or fixed for immunocytochemistry and apoptosis assays using Hoechst staining. p63 manipulation was achieved by transfecting cells with the overexpression plasmids TAp63γ and TAp63γ mutant that lack p63 transactivation activity (kind gift from Dr. Kurt Engeland, University of Leipzig), or 100 nM of siRNA designed to knockdown mouse p63 expression (M-040654-01, Dharmacon, Waltham, MA, USA). As a control, cells were transfected with the corresponding empty vector plasmid (pcDNA3.1), or control siRNA containing a scrambled sequence that does not lead to specific degradation of any known cellular mRNA. Transfection efficiencies were assessed by immunoblotting analyses and found to be ∼70% for JMJD3 and 80% for TAp63γ overexpression plasmids. p63 protein levels, in turn, decreased by ∼60% after transfection with siRNAs. In experiments using the protein synthesis inhibitor CHX, cells were previously transfected with TAp63γ and either Flag-JMJD3 or Flag-JMJD3 mutant plasmids 24 h prior to addition of 50 μg/mL CHX (Sigma-Aldrich Co.), and maintained for 4, 6 and 8 h.

### Total, Cytosolic and Nuclear Protein Extraction

For total protein extracts, NSCs were lysed in ice-cold buffer (10 mM Tris-HCl, pH 7.6, 5 mM MgCl_2_, 1.5 mM potassium acetate, 1% Nonidet P-40, 2 mM dithiothreitol) and protease inhibitor cocktail tablets Complete (Roche Applied Science, Mannheim, Germany) for 30 min, and then sonicated. The lysate was centrifuged at 3,200 g for 10 minutes at 4°C, and the supernatant recovered. For nuclear and cytosolic extracts, cells were lysed with hypotonic buffer (10 mM Tris-HCl, pH 7.6, 5 mM MgCl_2_, 1.5 mM potassium acetate, 2 mM dithiothreitol) and protease inhibitors, homogenized with 40 strokes in a loose fitting Dounce, and centrifuged at 500 g for 10 min at 4°C. Cytosolic proteins were recovered in the supernatant, while the nuclear pellet was washed in buffer containing 10 mM Tris-HCl, pH 7.6, 5 mM MgCl_2_, 0.25 M sucrose, 0.5% Triton X-100 and protease inhibitors, then resuspended and sonicated in buffer containing 10 mM Tris-HCl, pH 7.6, 0.25 M sucrose with protease inhibitors. Finally, the suspension was centrifuged through 0.88 M sucrose at 2,000 g for 20 min at 4°C, and nuclear proteins were recovered in the supernatant.

### Immunoblotting

Protein levels of p63, JMJD3, Flag, β-III tubulin, NeuN, MAP2 and H3K27me3 were determined by Western blot analysis in 8 or 12% sodium dodecyl sulphate polyacrylamide gel electrophoresis, using either primary mouse monoclonal antibodies reactive to p63 (4A4; Santa Cruz Biotechnology, Santa Cruz, CA), NeuN (MAB377; Chemicon International, Temecula, CA), Flag (M2; Sigma-Aldrich Co.), and β-III tubulin (Tuj1; Covance, Princeton, New Jersey), or primary polyclonal antibodies reactive to MAP2 (AB5622, Chemicon International) H3K27me3 (Abcam plc, Cambridge, UK) and JMJD3 (RB10082; Abgent Inc, San Diego, CA), as well as corresponding secondary antibodies conjugated with horseradish peroxidase (Bio-Rad Laboratories, Hercules, CA, USA). Membranes were processed for protein detection using Super Signal^TM^ substrate (Pierce, Rockford, IL, USA). β-Actin (AC-15; Sigma-Aldrich Co.) was used as loading control. GAPDH (6C5; Santa Cruz Biotechnology) and total histone H3 (Millipore Corporation, Temecula, CA, USA) were used as markers for cytoplasmic and nuclear protein extraction, respectively. Protein concentrations were determined using the Bio-Rad protein assay kit (Bio-Rad Laboratories), according to the manufacturer's specification.

### Immunocytochemistry

NSCs undergoing differentiation were fixed with 4% paraformaldehyde in phosphate-buffered saline (PBS) for 30 min. Cells were then blocked for 1 h at room temperature in PBS containing 0.1% Triton-X-100, 1% FBS, and 10% normal donkey serum (Jackson Immuno Research Laboratories, Inc., West Grove, PA). Subsequently, cells were incubated with either primary monoclonal antibody reactive to p63 (4A4; 1∶50) or primary polyclonal antibody reactive to JMJD3 (KDM6B; 1∶50) in blocking solution, overnight at 4°C. After three washes with PBS, cells were incubated with anti-rabbit Alexa Fluor 594- or anti-mouse Alexa Fluor 568-conjugated secondary antibodies (Invitrogen Corp.; 1∶200) for 2 h at room temperature. In control samples, the primary antibody was replaced by blocking buffer. Cells were incubated with Hoechst dye for nuclear staining. Images were acquired with an Axioskop fluorescence microscope (Carl Zeiss GmbH, Hamburg, Germany).

### Immnunoprecipitation Assay

The physical association between p63 and JMJD3 was evaluated by immunoprecipitation analysis. In brief, whole-cell extracts were prepared by lysing cells by means of sonication in lysis buffer (50 mM Tris-HCl pH 7.4, 180 mM NaCl, 1 mM EDTA, 0,5% Triton X-100) and protease inhibitors (Roche Applied Science). Immunoprecipitation experiments were carried out using the monoclonal antibody reactive to p63 (4A4) and the Ezview Red Protein G Affinity Gel (Sigma-Aldrich Co.). Typically, 500 μg of lysate was incubated with 1 μg of p63-specific antibody overnight at 4°C. Immunoblots were then probed with the rabbit polyclonal antibody reactive to JMJD3 (KDM6B). p63 protein levels were determined in the same membrane after stripping off the immune complex for the detection of JMJD3. Immunoprecipitation assays using mouse monoclonal antibodies reactive to IgG showed neglectable binding with either JMJD3 or p63. Methylated levels of p63 were detected by immunoprecipitation analysis in denaturing conditions. In brief, whole-cell extracts were prepared by lysing cells by means of sonication in lysis buffer (50 mM Tris-HCl pH 7.4, 180 mM NaCl, 1 mM EDTA, 1% Triton X-100, 10 mM dithiothreitol) and protease inhibitors (Roche Applied Science). Immunoprecipitation experiments were carried out using the monoclonal antibody reactive to p63 and the Ezview Red Protein G Affinity Gel (Sigma-Aldrich Co.). Typically, 500 μg of lysate was incubated with 1 μg of p63-specific antibody overnight at 4°C. Immunoblots were then probed with the rabbit polyclonal methylated lysine (MeK) (Abcam plc) antibody. p63 expression was determined in the same membrane after stripping off the immune complex for the detection of MeK. Finally, the results of MeK after p63 immunoprecipitation were normalized with those obtained using mouse monoclonal antibodies reactive to IgG immunoprecipitation assays as well as with p63 total levels.

### Evaluation of Apoptosis

Hoechst labeling of NSCs were used to detect apoptotic nuclei. In brief, for morphologic evaluation of apoptosis, medium was gently removed to minimize detachment of cells. Attached cells were fixed with 4% paraformaldehyde in PBS, pH 7.4, for 10 minutes at room temperature, incubated with Hoechst dye 33258 (Sigma-Aldrich Co.) at 5 μg/ml in PBS for 5 minutes, washed with PBS and mounted using Fluoromount. Fluorescent nuclei were scored blindly and categorized according to the condensation and staining characteristics of chromatin. Normal nuclei showed noncondensed chromatin dispersed over the entire nucleus. Apoptotic nuclei were identified by condensed chromatin, contiguous to the nuclear membrane, as well as nuclear fragmentation of condensed chromatin.

### Densitometry and Statistical Analysis

The relative intensities of protein bands were analyzed using the QuantityOne Version 4.6 densitometric analysis program (Bio-Rad Laboratories) and normalized to the respective loading controls. Statistical analysis was performed using GraphPad InStat version 3.00 (Graph-Pad Software, San Diego, CA, USA) for the analysis of variance and Bonferroni's multiple comparison tests. Values of *p*<0.05 were considered significant.

## Results

### TAp63γ and JMJD3 Levels Increase Throughout Mouse NSC Differentiation

**Figure 1:**
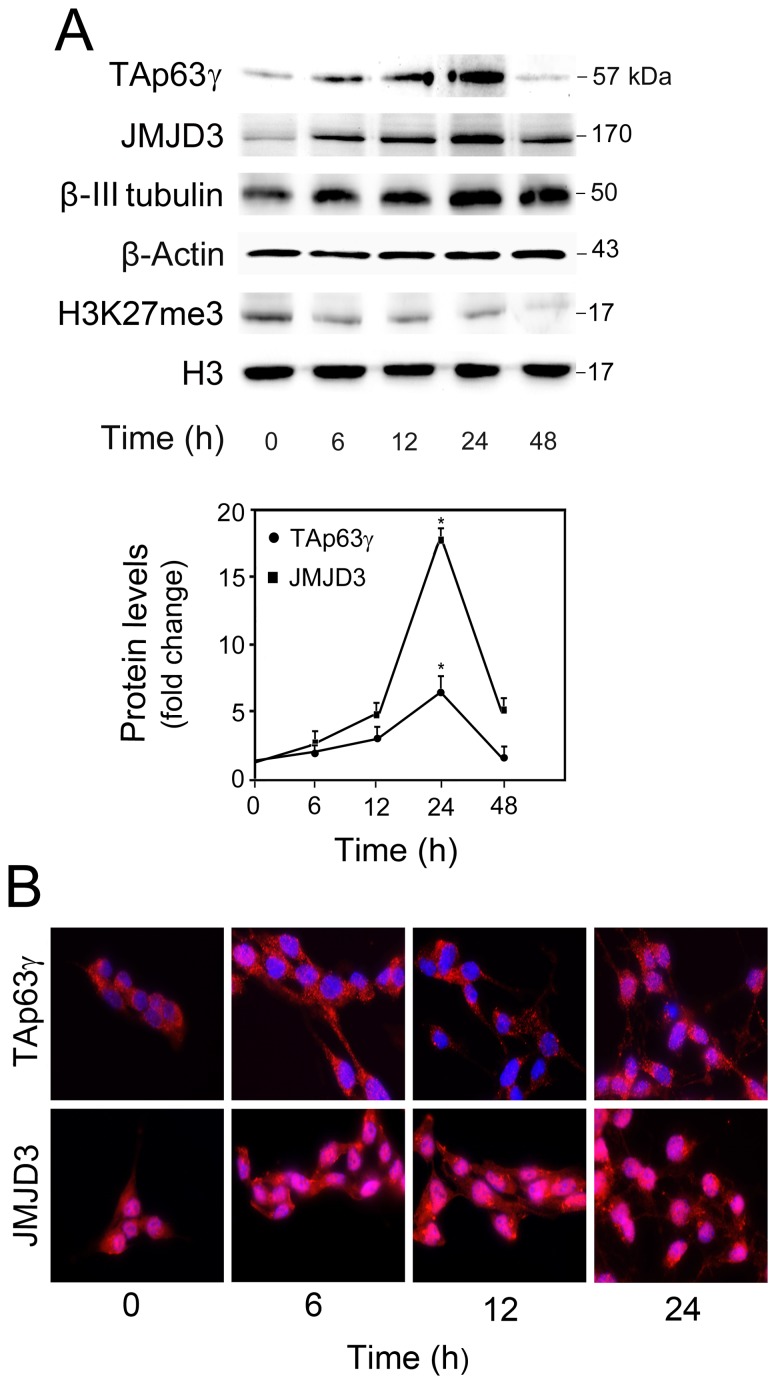
Endogenous TAp63γ protein levels increase at early stages of neural differentiation. Undifferentiated (0 h) and differentiated mouse NSCs were collected for Western blot analysis or fixed for immunostaining at indicated time-points, as described in Materials and Methods. (A) Protein levels of TAp63γ, JMJD3, β-III tubulin, and H3K27me3 throughout NSC differentiation. Representative immunoblots (top), and TAp63γ and JMJD3 protein levels (bottom). β-Actin and H3 were used as loading controls. Immunoblots are representative of at least three different experiments. TAp63γ and JMJD3 protein levels were normalized to endogenous β-Actin protein levels and expressed as mean ± SEM for at least three different experiments. *p<0.01 from control, undifferentiated. (B) Fluorescence staining of TAp63γ and JMJD3 throughout NSC differentiation. Hoechst dye was used for nuclear staining. Images are representative of at least 3 different experiments. Scale bar  = 10 µm.

Several lines of evidence point toward the involvement of classical apoptotic molecules in neural differentiation, including p53, caspases and Bcl-2 family members. To investigate p63 involvement in neurogenesis, we first evaluated endogenous p63 levels throughout neural differentiation of NSCs. Comparing with the exogenous overexpression of several p63 isoforms [3], TAp63γ (57 kDa) was the only endogenous isoform detected by Western blot analysis in NSCs. Importantly, TAp63γ levels increased by 7-fold at 24 h of NSC differentiation (Fig. 1A). JMJD3 upregulation was observed as early as at 6 h of differentiation, peaking at 24 h (Fig. 1A), in agreement with previous studies [28]. Accordingly, JMJD3 activity was also increased during differentiation, as demonstrated by the reduced levels of H3K27 trimethylation throughout time (Fig. 1A). The neuronal marker β-III tubulin increased throughout differentiation, peaking at 24 h (Fig. 1A), as previously described [11]. Other neural markers, including mouse achaete scute homolog-1 (Mash1) and neuronal differentiation 1 (NeuroD1) were also markedly increased (Fig. S1). Immunocytochemistry analysis corroborated Western blot data by showing upregulation and nuclear distribution of TAp63γ and JMJD3 at 24 h of differentiation (Fig. 1B). These results suggest that JMJD3 and TAp63γ are coordinately regulated to influence neural-specific gene expression programs.

### JMJD3 Regulates TAp63γ Protein in a Demethylase Activity-dependent Manner

**Figure 2:**
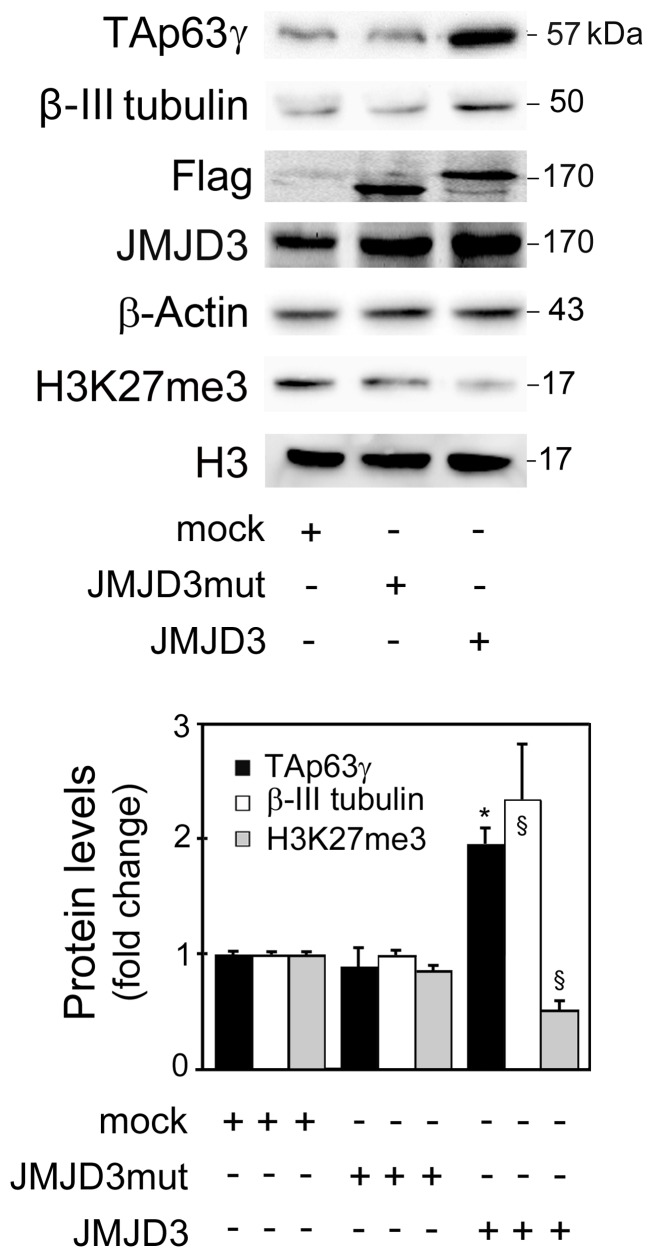
JMJD3 modulates TAp63γ levels during neural differentiation. Mouse NSCs undergoing differentiation were transfected with JMJD3 or JMJD3 mutant (JMJD3mut), overexpression plasmids or no plasmid (mock), and collected for Western blot analysis as described in Material and Methods. Representative immunoblots of TAp63γ, β-III tubulin, Flag, JMJD3 and H3K27me3, in control (mock), JMJD3mut- and JMJD3-overexpressing cells (top) and respective protein levels (bottom). β-Actin and H3 were used as loading controls. Immunoblots are representative of at least three different experiments. TAp63γ and β-III tubulin protein levels were normalized to endogenous β-Actin whereas H3K27me3 protein levels were normalized to endogenous total H3, when compared with mock cells. Results were expressed as mean ± SEM for at least three different experiments. §*p*<0.05 and *p<0.01 from control.

To clarify whether JMJD3 regulates TAp63γ during neurogenesis, we overexpressed JMJD3 by transfecting NSCs with a Flag-JMJD3 plasmid and evaluated TAp63γ protein levels 24 h after transfection. In control experiments, cells were transfected with either a Flag-JMJD3 mutant plasmid that does not contain the C-terminal region associated with JMJD3 demethylase activity, or no plasmid (mock). Overexpression of JMJD3 markedly increased TAp63γ protein levels when compared with control (mock) cells (*p*<0.01) (Fig. 2). To better distinguish endogenous JMJD3 protein from the transfected versions, we have also detected Flag using a Flag antibody. Of note, overexpressing the mutant form of JMJD3 had no effect on TAp63γ protein levels, indicating that JMJD3-induced increase of TAp63γ is dependent on its demethylase activity. Consistent with a role for JMJD3 as H3K27 demethylase, JMJD3 overexpression resulted in ∼50% decreased levels of trimethylated H3K27 when compared with mock or cells transfected with JMJD3 mutant plasmid (*p*<0.05) (Fig. 2). As expected, JMJD3 overexpression significantly enhanced the neuronal marker β-III tubulin by ∼2-fold when compared with mock- and JMJD3 mutant plasmid-transfected cells (*p*<0.05) (Fig. 2). These results are consistent with a role for JMJD3-dependent demethylation in neurogenesis progression. Moreover, the data here presented suggest that JMJD3-demethylase activity modulates TAp63γ protein levels and this may account, at least in part, for the neuronal commitment of NSCs.

### TAp63γ Manipulation Influences Neuronal Differentiation

**Figure 3:**
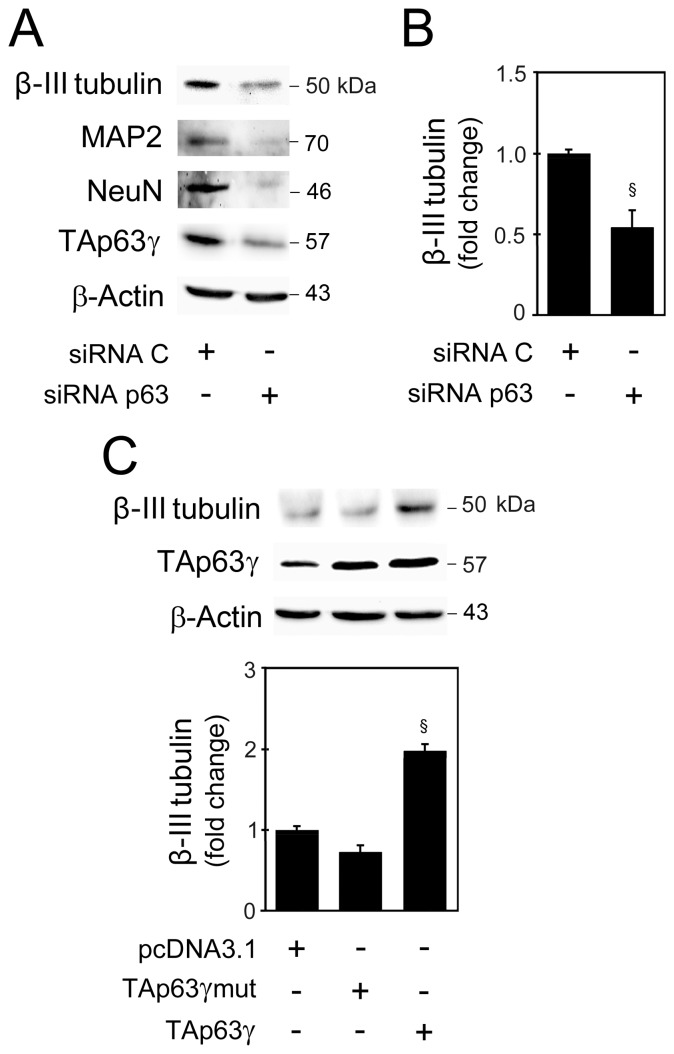
Modulation of TAp63γ levels in mouse NSCs affects neuronal differentiation. Mouse NSCs undergoing differentiation were transfected with p63 siRNA or TAp63γ overexpression plasmids and collected for Western blot analysis as described in Material and Methods. (A) Representative immunoblot of β-III tubulin, NeuN, MAP2 and TAp63γ in cells transfected with 100 nM of either p63 siRNA or control siRNA (siRNA C) for 24 h. (B) Respective β-III tubulin protein levels. (C) Protein levels of β-III tubulin and TAp63γ in cells transfected with overexpression plasmids TAp63γ, TAp63γ mutant (TAp63γmut) or its corresponding empty vector (pcDNA3.1). Representative immunoblot of β-III tubulin and TAp63γ (top) and respective β-III tubulin protein levels (bottom). Results were normalized to endogenous β-Actin protein levels and expressed as mean ± SEM for at least three different experiments. §*p* <0.05 from control.

To substantiate a role for TAp63γ in non-embryonic neuronal differentiation, we tested the functional relevance of TAp63γ in regulating β-III tubulin abundance in NSCs undergoing differentiation by short-interference RNA (siRNA)-mediated gene knockdown of p63. Immunoblot analysis revealed that knockdown of TAp63γ by specific p63 siRNA resulted in a ∼50% reduction in β-III tubulin levels when compared with control siRNA-transfected cells (Fig. 3A and B). Other neural markers, including the neuronal nuclei (NeuN) and microtubule-associated protein 2 (MAP2) were also markedly reduced (Fig. 3A). These results were corroborated by immunofluorescence analysis (data not shown) and indicate that the increase in TAp63γ throughout NSC differentiation is important for neuronal fate specification. In addition, overexpression of TAp63γ significantly increased β-III tubulin protein levels by ∼2-fold when compared with cells transfected with the corresponding empty vector (*p*<0.01) (Fig. 3C). Importantly, overexpression with the mutant form of TAp63γ that lacks p63 transactivation activity had no effect on β-III tubulin protein levels (Fig. 3C), indicating that TAp63γ-mediated increase of β-III tubulin is dependent on its transactivation activity.

### TAp63γ is Directly Demethylated by JMJD3 during Neural Differentiation

**Figure 4:**
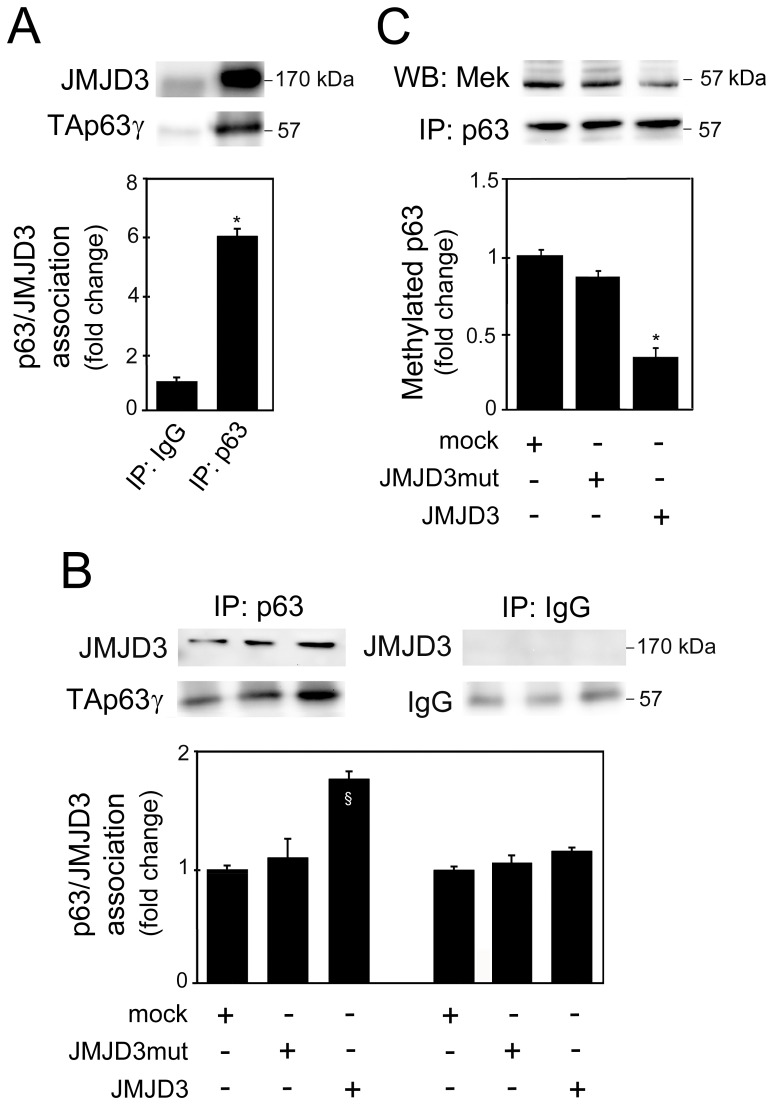
TAp63γ and JMJD3 directly associate during neural differentiation. Mouse NSCs undergoing differentiation were collected and processed for immunoprecipitation assays with either p63 antibody or mouse IgG as control, as described in Material and Methods. (A) Representative immunoblot of JMJD3 and TAp63γ (top) and histogram of TAp63γ/JMJD3 endogenous association (bottom). (B) Representative immunoblot of JMJD3 and TAp63γ (top) and histogram of TAp63γ/JMJD3 association (bottom) in cells transfected with JMJD3 or JMJD3 mutant (JMJD3mut) overexpression plasmids, or no plasmid (mock). (C) Representative immunoblot of methylated lysine (MeK) and TAp63γ (top), and histogram of TAp63γ methylated levels (bottom). All densitometry values for JMJD3 and methylated lysines were normalized to the respective p63 expression, and the results expressed as mean ± SEM for at least three different experiments. §*p*<0.05 and *p<0.01 from controls. IP, Immunoprecipitation; WB, Western blot; MeK, Methyl K pan.

Since JMJD3 also demethylates non-histone proteins, including p53 [9], we hypothesized that JMJD3 may possibly demethylate TAp63γ during neurogenesis. Thus, the interaction between JMJD3 and TAp63γ, as well as JMJD3-mediated TAp63γ demethylation were investigated by immunoprecipitation assays. Specifically, to evaluate TAp63γ and JMJD3 endogenous association during differentiation of NSCs, we immunoprecipitated TAp63γ from total protein extracts with p63 antibody, and immunoblots were probed with JMJD3 antibody. As depicted in Fig. 4A, JMJD3 directly binds to TAp63γ during NSC differentiation (*p*<0.01). To investigate whether TAp63γ/JMJD3 association is dependent on JMJD3-demethylase activity, TAp63γ immunoprecipitation was performed in NSCs overexpressing either JMJD3 wild-type or JMJD3 mutant plasmids. Western blot analysis revealed a significant increase in TAp63γ/JMJD3 association in cells overexpressing JMJD3 wild-type when compared with mock (*p*<0.05) (Fig. 4B). Importantly, the association between TAp63γ and JMJD3 was not increased in cells transfected with mutant JMJD3, suggesting that the interaction between TAp63γ and JMJD3 is dependent on the catalytic JmjC domain of JMJD3. To investigate whether TAp63γ/JMJD3 association results in modification of TAp63γ methylation status, TAp63γ immunoprecipitation under denaturating conditions was performed in NSCs transfected with JMJD3 wild-type or JMJD3 mutant overexpression plasmids. Subsequent detection of methylated TAp63γ was performed by probing immunoblots with a pan-methylated lysine antibody. Notably, TAp63γ was significantly less methylated in cells overexpressing JMJD3 wild-type, as compared to cells transfected with the C-terminal mutant form of JMJD3 or control (mock) cells (*p*<0.01) (Fig. 4C). These results suggest that the proneuronal effects of TAp63γ during differentiation of NSCs might involve direct JMJD3-dependent demethylation.

### TAp63γ is Stabilized by JMJD3-demethylase Activity and Accumulates in the Nucleus

**Figure 5:**
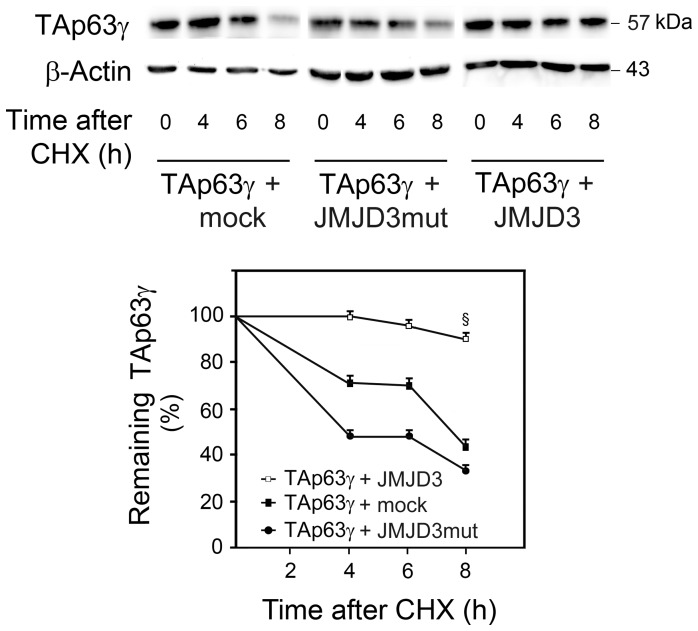
TAp63γ is stabilized by JMJD3 during mouse NSC differentiation. Cells co-transfected with TAp63γ and with the overexpression plasmids JMJD3 (TAp63 + JMJD3), JMJD3 mutant (TAp63 + JMJD3mut), or no plasmid (TAp63 + mock) were untreated (0 h) or treated with 50 μg/mL CHX for 2, 4, 6, and 8 h. At the indicated time-points, total proteins were extracted for immunoblot analysis as described in Materials and Methods. Representative immunoblots of TAp63γ after CHX treatment (top) and percentage of remaining TAp63γ after CHX treatment (bottom). Results were normalized to endogenous β-Actin protein levels and expressed as mean ± SEM for at least three different experiments. §*p*<0.05 from control.

**Figure 6:**
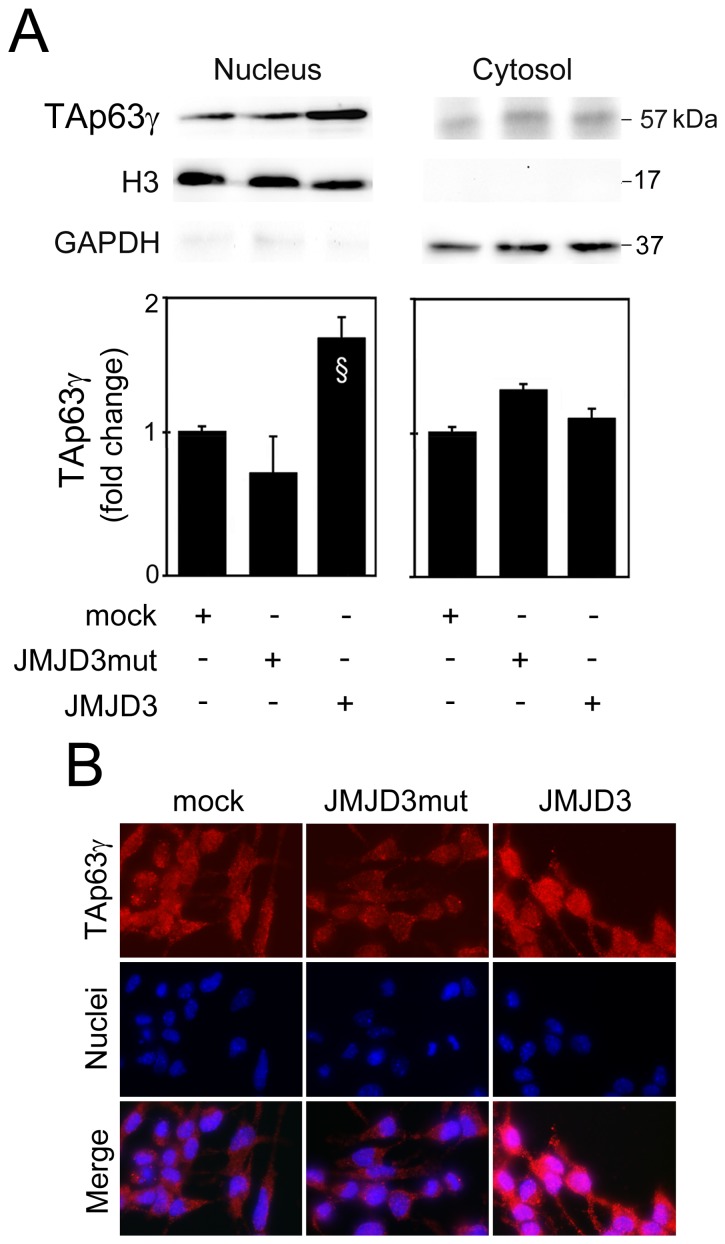
TAp63γ is translocated to the nucleus in a demethylase activity-dependent manner. Mouse NSCs undergoing differentiation were transfected with JMJD3 or JMJD3 mutant (JMJD3mut) overexpression plasmids, or no plasmid (mock), and collected for Western blot analysis, immunocytochemistry and apoptosis assays as described in Material and Methods. (A) Representative immunoblots of nuclear and cytosolic TAp63γ (top) and respective protein levels (bottom). GAPDH and H3 were used as loading controls for cytosolic and nuclear fractions, respectively. Results were normalized to the respective endogenous controls and expressed as mean ± SEM for at least three different experiments. §*p*<0.05 from control. (B) Subcellular localization of TAp63γ in transfected mouse NSCs. Hoechst dye was used for nuclear staining and morphologic evaluation of apoptosis. Images are representative of at least 3 different experiments. Scale bar  = 10 µm.

p63 activity is controlled by an intricate network of PTM that targets and modulates its transcriptional activity, stability or subcellular trafficking [29]. These PTM are catalyzed by a large number of enzymes that contribute to the activation of p63 through a variety of mechanisms. To investigate the effect of JMJD3-mediated TAp63γ demethylation on TAp63γ protein stability, we evaluated TAp63γ half-life in the presence and absence of JMJD3-demethylase activity. For this purpose, NSCs co-transfected with TAp63γ and either Flag-JMJD3, Flag-JMJD3 mutant or no plasmid (mock) were treated with the protein synthesis inhibitor CHX. Our results demonstrate that TAp63γ half-life was reduced to less than 8 and 4 h, respectively in control (mock)- and mutant JMJD3-transfected cells (*p*<0.05). In contrast, TAp63γ protein levels only slightly decreased in the presence of JMJD3 wild-type (Fig. 5), showing that JMJD3-demethylase activity indeed regulates the stability of TAp63γ. We next investigated the effect of JMJD3-mediated TAp63γ demethylation on TAp63γ subcellular distribution (Fig. 6). Increased nuclear localization of TAp63γ was observed in NSCs overexpressing wild-type JMJD3 compared with control (mock)- and mutant JMJD3-transfected cells (*p*<0.05) (Fig. 6A). Immunocytochemistry analysis confirmed Western blot data by showing TAp63γ accumulation in the nucleus of JMJD3-transfected cells compared to cells overexpressing either the mutant form of JMJD3 or control (mock) cells (Fig. 6B). Together, these results suggest that TAp63γ demethylation by JMJD3 is crucial for its stabilization and accumulation in the nucleus. Moreover, these data support the hypothesis that JMJD3-induced TAp63γ nuclear translocation might direct NSCs to a neuronal fate specification, through the transcriptional activation of proneuronal genes. Importantly, TAp63γ nuclear accumulation was not associated with an increase in apoptotic cell death, as confirmed by Hoechst staining of nuclei (Fig. 6B).

## Discussion

This study demonstrates for the first time a role for p63 in adult neural progenitors, directing NSCs to a neuronal phenotype. Our findings are in accordance with postnatal p63 expression in the subventricular zone of the lateral ventricle [19], a region for continued adult neurogenesis. In contrast with the lack of p63 effect on central nervous system development [19], [20], our results point toward an age-dependent p63 involvement in the regulation of neural differentiation. Furthermore, we demonstrate that, during neural differentiation, p63 interacts with JMJD3, a key regulator of neurogenesis [30], which results in the modulation of p63 methylation status. Finally, our results demonstrate that JMJD3-demethylase activity regulates p63 half-life and nuclear accumulation during mouse NSC differentiation.

Much attention has been centered on p63 regulatory pathways in epidermal differentiation, due to the severe epithelial phenotype of p63 null mice [18]. Recently, a role for p63 on cardiac differentiation of embryonic stem cells has also been reported [31]. However, the precise function for p63 during neural differentiation remains largely unknown, when compared with other members of the p53 family. In fact, p53 and p73 are well-established key regulators of both embryonic and adult neurogenesis. Mice lacking p53 display elevated proliferation rate in the neurogenic niche of the adult lateral ventricle wall, as well as increased self-renewal of *in vitro* propagated p53 −/− NSCs [13]. Moreover, a relevant role for p53 in the biology of NSCs derived from the embryo olfactory bulb has been identified, where lack of p53 increases neurosphere-forming potential of precursor cells by favoring self-renewal capacity [14]. TAp73, in turn, is an essential regulator of stemness in NSCs, which maintains an adequate neurogenic pool by promoting self-renewal and proliferation and inhibiting premature senescence of NSC and early progenitor cells, in both embryonic and adult neurogenesis [32]. In addition, the width of neurogenic areas appears to be significantly reduced in brains of embryonic and adult p73 knockout mice [33]. Curiously, TAp73 acts via the basic helix-loop-helix (bHLH) Hey2 to promote long-term maintenance of neural precursors [34]. p73 deficiency results in impaired self-renewal and premature neuronal differentiation of mouse neural progenitors independently of p53 [35].

In contrast with p53 and p73, there is still a breach in the literature regarding p63 potential involvement in adult neurogenesis by cell death-independent mechanisms. Further, emerging knowledge suggests that a number of apoptosis-associated factors, including p53, regulate neural differentiation [18], [31]. This led us to explore p63 involvement in adult mouse NSC differentiation, which are capable of tripotential differentiation in neurons, glial cells and oligodendrocytes [25], resembling adult neurogenesis. Several studies have claimed that p63 is dispensable for embryonic neurogenesis. However, since p63 knockout mice do not survive into postnatal life, a role for p63 in the adult brain cannot be excluded.

In this study, a detailed characterization of p63 isoform expression [3] revealed that only one isoform with ∼57 kDa was detected and subsequently confirmed as TAp63γ by comparison with exogenous p63 isoform overexpression [5]. Time-course analysis of TAp63γ protein levels throughout NSC differentiation demonstrated an increase of TAp63γ at early stages of neural differentiation, when JMJD3 protein levels and activity were already significantly increased. Accordingly, we have previously demonstrated that JMJD3 expression and activity at the Hoxc8 promoter increased early in mouse NSC differentiation [9]. Importantly, overexpression of JMJD3 in NSCs undergoing differentiation resulted in increased levels of TAp63γ protein, accompanied by increased neuronal marker β-III tubulin. We have previously reported a similar effect of JMJD3 on the neural precursor Pax6 in JMJD3-transfected mouse NSCs [9]. All together, these results suggest that JMJD3 modulates TAp63γ levels during neural differentiation, which might contribute to the neuronal fate specification of NSCs. In accordance with this hypothesis, overexpression of TAp63γ during early differentiation of NSCs resulted in increased β-III tubulin levels, whereas downregulation of TAp63γ expression by specific p63 siRNA abrogated the increase in β-III tubulin.

Interestingly, in NSCs undergoing differentiation, both TAp63γ and β-III tubulin were modulated by JMJD3 in a demethylase activity-dependent manner. While not excluding an effect of H3K27 demethylation on NSC neuronal differentiation, our results also suggest that TAp63γ-mediated neuronal differentiation essentially relies on TAp63γ direct demethylation by JMJD3. In this respect, JMJD3 was reported to bind non-histone proteins, including p53 [9], much like other demethylases [36]. Our results demonstrate a direct interaction between TAp63γ and JMJD3, dependent of the C-terminal region of JMJD3, and modulation of TAp63γ methylation levels by JMJD3. Importantly, JMJD3-demethylase activity appears to be relevant in regulating TAp63γ protein half-life. Cycloheximide experiments demonstrated that modulation of TAp63γ levels by JMJD3 during differentiation of NSCs occurs through protein stabilization and not by transcriptional regulatory mechanisms. Supporting this idea, it has recently been shown that among several genes involved in neurogenesis and neuronal differentiation that are up-regulated by H3K27 demethylation, p63 is not included [37]. This suggests that JMJD3-mediated regulation of TAp63γ in NSCs does not occur at the transcriptional level. In addition to TAp63γ stabilization, we here show that JMJD3-demethylase activity increases TAp63γ nuclear accumulation during NSC differentiation. These results are in agreement with those previously obtained for JMJD3 and p53 in mouse NSCs [9], reinforcing the similarities in structural and function properties of p53 family members. Noteworthy, TAp63γ cellular distribution concurs with a role for this protein as a transcriptional activator of pro-neuronal gene expression. Accordingly, NSCs cells transfected with the mutant form of TAp63γ that lacks p63 transactivation activity failed to differentiate in β-III tubulin-positive cells, indicating that TAp63γ-mediated neuronal differentiation might be dependent on its transactivation activity. This study constitutes the first to show that TAp63γ demethylation affects its stability and subcellular localization. Similar effects were already described for p63 phosphorylation and acetylation, the best characterized p63 PTM [38] resulting in different cellular outcomes. A deepest knowledge of these regulatory modifications of p63 protein will improve our understanding of p63 function during adult neural differentiation. In this respect, future studies are warranted to determine the specific residues demethylated by JMJD3 and to address the importance of TAp63γ demethylation in neurogenesis by modification of the identified residues. Since most mutated p63 proteins found in patients suffering from ectodermal dysplasia syndromes [39] are more stable than wild-type peptides, it would be interesting to clarify whether the mutated residues are sites of methylation. We also anticipate TAp63γ protein demethylation to be highlighted as a PTM crucial for the neurogenic process.

In conclusion, our results clearly demonstrate that TAp63γ and JMJD3 directly interact during NSC differentiation, in a JMJD3 C-terminal region-dependent manner. In addition, both TAp63γ protein stability and nuclear accumulation appear to be modulated by JMJD3-demethylase activity during neural differentiation. These findings suggest that JMJD3 is a regulator of TAp63γ during NSC differentiation, and JMJD3-mediated TAp63γ demethylation plays a role in proneural gene expression.

## Supporting Information

Figure S1 **Mash1 and NeuroD1 mRNA levels increase during neural differentiation.** Cells were induced to differentiate and collected 72 h thereafter. Total mRNA was extracted for RT-PCR analysis. Histograms of total mRNA levels of Mash1 and NeuroD1 during mouse NSC differentiation. Results were normalized to the GAPDH mRNA expression and expressed as mean ± SEM for at least three different experiments. *p<0.01 from controls.
(TIF)
