# Supplementary figures and images for: TAp63γ Demethylation Regulates Protein Stability and Cellular Distribution during Neural Stem Cell Differentiation
Source: PLoS One. 2012 Dec 14;7(12):e52417. doi: 10.1371/journal.pone.0052417 (PMC3522631; doi:10.1371/journal.pone.0052417)

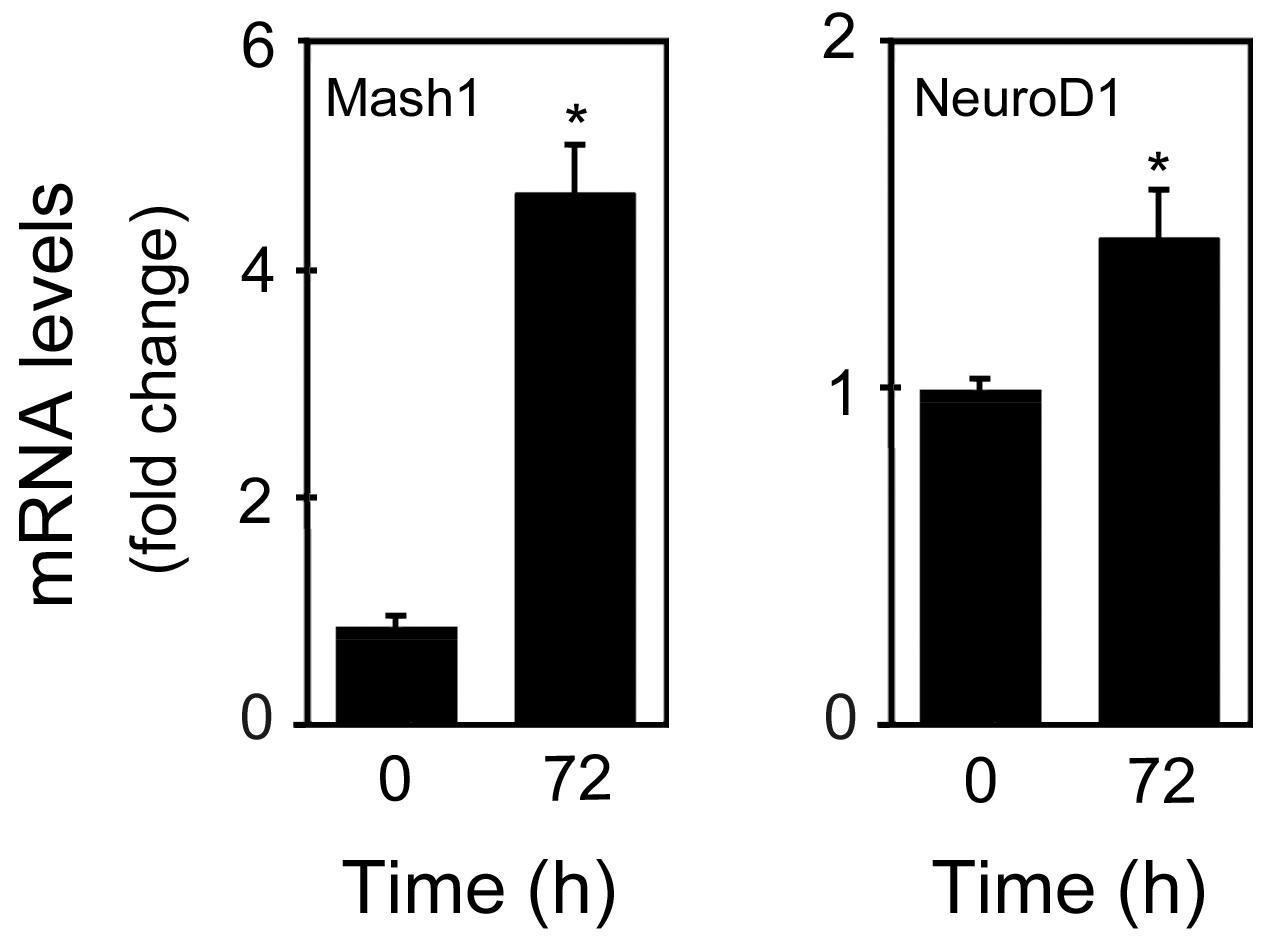

Supplement: Figure S1 — Mash1 and NeuroD1 mRNA levels increase during neural differentiation. Cells were induced to differentiate and collected 72 h thereafter. Total mRNA was extracted for RT-PCR analysis. Histograms of total mRNA levels of Mash1 and NeuroD1 during mouse NSC differentiation. Results were normalized to the GAPDH mRNA expression and expressed as mean ± SEM for at least three different experiments. *p<0.01 from controls. (TIF) [file pone.0052417.s001.tif]
